# Inequalities in Fertility‐Impacting Cancer Incidence Among Young Populations in the United States

**DOI:** 10.1002/cam4.70797

**Published:** 2025-04-06

**Authors:** Katherine I. Tierney, Jennifer Therrien, Stephanie Ellwood, Lisa Graves

**Affiliations:** ^1^ Department of Sociology Western Michigan University Kalamazoo Michigan USA; ^2^ School of Medicine Western Michigan University Homer Stryker MD Kalamazoo Michigan USA

**Keywords:** cancer, health disparities, infertility, late effects, social demography

## Abstract

**Introduction:**

Infertility is a concerning late effect of cancer and cancer treatments, yet referrals for fertility preservation are unequal across U.S. sociodemographic groups. Although all‐site cancer incidence varies across U.S. sociodemographic groups, it is unclear whether fertility‐impacting cancers, specifically, are unevenly distributed by sex or race/ethnicity.

**Methods:**

Cross‐sectional analysis of cancer registry data from the Surveillance, Epidemiology, and End Results (SEER) Program (2010–2020). Age‐specific demographic rates and negative binomial regression with an exposure for population size were employed to assess inequalities in the incidence rates of fertility‐impacting cancers among U.S. individuals aged 39 and younger. Wald tests were used to compare coefficients across the multivariable negative binomial regression models.

**Results:**

Women had higher incidence rates of fertility‐impacting cancers (cancers of the reproductive organs, cancers in areas proximal to the reproductive organs or that contribute to reproductive functioning, and other cancers identified in the literature as fertility‐impacting) in the fully adjusted models. These associations differed from the patterns observed among all other types of cancers. The incidence rates of fertility‐impacting cancers also varied by race/ethnic groups. However, the patterning observed by race‐ethnicity varied between the three fertility‐impacting cancer groups.

**Conclusion:**

The burden of fertility‐impacting cancers is unequal across sex and race/ethnic groups. The sociodemographic patterns observed in fertility‐impacting cancers differ substantively from cancers that were not identified as fertility‐impacting. The findings reinforce the importance of screening for fertility‐impacting cancers and identify a potential unmet need for both fertility preservation referrals among cancer patients and access to fertility treatment for survivors of cancer.

## 1 Introduction

Cancer and cancer treatments can have long‐term impacts on fertility. Research has shown lower odds of pregnancy, live birth, and conception along with delayed time to conception among cancer survivors [1, 2, 3, 4, 5, 6]. Women cancer survivors are also at risk of premature ovarian failure or early menopause [7]. In part, these outcomes are related to cancer treatments [8, 9]. Specifically, radiation therapy, cumulative alkylating agent dose, and other types of treatments have been shown to reduce fertility [1, 4, 9, 10, 11]. Additionally, bone marrow transplants, commonly used for the treatment of leukemia and other types of cancer, increase the risk for infertility [10, 12].

While these cancer treatments can impact fertility, specific sites of cancer also have direct and indirect impacts on fertility. Most directly, cancers of the reproductive organs and in areas proximal to these organs can impact reproductive functioning [8, 13, 14, 15]. Similarly, cancers that impact the brain, thyroid, and other areas of hormone production and regulation (e.g., endocrine cancers, central nervous system tumors, hypothalamus and pituitary gland cancers) can have direct or indirect effects on fertility [8]. In addition, prior research has also found associations between infertility and childhood cancers, including hematologic malignancies, Wilms tumor, malignant bone tumors, and rhabdomyosarcomas [9] as well as other types of cancer such as Hodgkin's disease, leukemia, and lymphoma [5, 8, 13, 16]. Further, malignancy itself can also indirectly affect fertility through malnutrition, vitamin deficiency, and other factors [8]. These associations between infertility, cancer and cancer treatments have been documented across country contexts (e.g., [4, 5, 6, 11, 16, 17]).

Given these associations between fertility and cancer/cancer treatment, fertility preservation (FP) is recommended by the American Society of Clinical Oncology for cancer patients in their reproductive years [18]. Although FP counseling and treatment are an important part of cancer care for patients [19], FP referrals are inconsistent and uneven across sociodemographic groups in the United States [20, 21, 22]. For example, studies in the United States have shown that referrals to FP are lower among patients with lower socioeconomic status (SES) and women, while referrals are higher among women who are white, older, and nulliparous [10, 20, 23, 24, 25, 26]. A small but growing body of research has identified a number of potential mechanisms for these differential FP referral patterns in the U.S., including (1) geographic access to FP, (2) provider knowledge, discomfort, and perceptions of FP, (3) patient prognosis, (4) uneven patient knowledge/education, (6) patient perceptions of FP and its costs [22, 27, 28, 29, 30, 31, 32].

Research has also shown that utilization of fertility treatments and fertility treatment‐seeking among U.S. women survivors of cancer are stratified. Specifically, women cancer survivors who seek fertility treatment are more likely to be married, white, college‐educated, nulliparous, younger, heterosexual, and to have higher incomes [33, 34, 35, 36]. Such inequalities in fertility treatment‐seeking are similar to the inequalities observed in the general U.S. population for women and men (for reviews see: [37, 38, 39]). In the general U.S. population, a number of potential mechanisms have been suggested for inequalities in fertility treatment‐seeking including economic access, provider biases, life‐course factors (e.g., reproductive history and desires), perceptions and attitudes of both infertility and fertility care, infrastructural barriers, and geographic barriers [39, 40, 41]. While some of these mechanisms likely contribute to inequalities among cancer survivors, it is also possible that differential FP referral rates and other experiences specific to this population create, mediate, or otherwise change the patterning in fertility care utilization.

One potential explanation for inequalities in both FP referrals among cancer patients and fertility treatment‐seeking among cancer survivors could be the differential need for services. That is, inequalities in fertility‐impacting cancer incidence rates could shape FP referrals and, down‐stream, fertility treatment utilization (in the general U.S. population, differential need *does not* explain disparities in fertility treatment use; the prevalence of infertility is higher or similar among women of color and women with lower SES, though these groups seek and receive care for infertility less often [42, 43, 44, 45]). Yet, although cancer incidence rates do vary across sociodemographic groups for a variety of social, behavioral, and biological reasons in the U.S. [46, 47], it remains unclear whether fertility‐impacting cancers as a group are evenly distributed across sociodemographic groups.

Thus, the aim of this study was to investigate the incidence rates of fertility‐impacting cancers across sex and racial/ethnic groups in the United States. There are widespread and well‐documented inequalities in health and health care utilization by both race/ethnicity and socioeconomic status in the United States (for reviews: [48, 49, 50, 51, 52, 53, 54]). The causes of these inequalities are rooted in the sociohistorical context of the United States and have been investigated elsewhere (e.g. [50, 55]. Therefore, the extent to which the inequalities observed in the United States align with other contexts may vary widely). Importantly, this paper does not seek to directly investigate the association between fertility‐impacting cancers and referrals to or use of FP. Instead, the present study is more broadly focused on investigating whether, and to what extent, there are inequalities in the incidence of fertility‐impacting cancers as a group. In the discussion, the paper compares and contrasts the results of our analyses with the existing patterns in FP referrals and highlights the importance of the findings for research and practice.

## 2 IRB Statement

This study was reviewed and deemed exempt by the IRB at Western Michigan University (IRB‐2023‐163).

## 3 Data

### 3.1 Surveillance, Epidemiology, and End Results (SEER) Program Cancer Registry Database

The data come from cancer registry data compiled from across the U.S. by the National Cancer Institute's SEER Program. The study used the cancer case counts from the SEER Research Limited‐Field Data released in November 2022 that includes 22 registries [56]. This publicly available registry database covers 47.9% of the U.S. population, which is the largest available coverage of all SEER Program registries [57]. This release of the SEER registry database includes a total of 16,683,417 cancer cases reported between 2000 and 2020.

The dataset includes information on sociodemographic characteristics, including age at diagnosis, race/ethnicity, and sex, along with detailed information about the type, location, and stage of the tumor, and types of cancer treatments received. The database does not include information on SES (e.g., education or income). Further details on the available variables and coding can be found in the documentation from the SEER Program website [58].

The SEER Program databases also include population counts matched to the populations in the registry data. To estimate the population at‐risk in our analyses, we used the “Populations—Total U.S. (1990–2020) <Katrina/Rita Adjustment>” dataset with a restriction to include only the population counts within the 22 registries included [59].

Both the cancer cases and population counts were restricted to (1) the geographic regions included in the cancer registry database, (2) populations aged 39 years and under (as the focus of this study was on fertility), and (3) the years 2010–2020. With these age and year restrictions in place, our dataset included 576,631 individual cancer cases. The authors of this paper were not involved in the collection of these data.

## 4 Measures and Methods

### 4.1 Outcome: Fertility‐Impacting Cancers

The central aim of this paper was to explore the incidence rates of fertility‐impacting cancers among populations 39 years of age or under. Thus, we used the ICD‐O‐3 codes in the SEER registry dataset to conceptually group cancers into four groups, including: (1) cancers of the reproductive organs (reproductive cancers), (2) cancers in locations close to the reproductive organs or areas that control hormone production relevant to fertility, with radiation and/or surgery in these areas confirmed (proximal plus cancers), (3) other cancers associated with fertility issues in the literature (literature‐identified cancers), and (4) all other types of cancer (a residual category).

The purpose of creating this typology of fertility‐impacting cancers was to, first, identify specific types of cancers that have higher potential to impact fertility due to location, due to impact on hormone production, and/or based upon previously conducted empirical research, and to, second, delineate specifically between fertility‐impacting cancers that may have more or less direct or indirect effects on future fertility. For example, cancers of the reproductive organs pose a direct risk to future fertility due to location [8, 60, 61, 62, 63, 64, 65]. However, brain cancer, as one example, may have less direct impacts on the reproductive organs, though may impact hormone production in a manner that impacts future fertility [62, 63, 66, 67]. Meanwhile, cancers in the residual category may have less impact on future fertility due to low rates among individuals of reproductive age, low survival rates, and/or less clear clinical pathways impacting reproduction.

Thus, with this proposed typology, this study is able to provide new information on whether and to what extent different types of fertility‐impacting cancers impact populations aged 39 and younger in the United States. These findings, in turn, allow for greater clarity into whether and to what extent these rates of fertility‐impacting cancers align with (or do not align with) fertility preservation referral patterns described above. In other words, comparing the patterning in all‐site cancer prevalence to the patterning in fertility preservation referrals for cancer patients may be misleading or imprecise due to the uneven potential impacts of different types of cancers on future fertility.

For the proximal plus cancers group, we combined information from the ICD‐O‐3 codes with the “RX Summ—Surg/Rad Seq” variable, which identified individuals who received radiation or surgery as part of their cancer care. Importantly, treatment reporting is limited in the SEER registries; it is not possible to distinguish between individuals who had no radiation/surgery and those with missing data [68]. Therefore, consistent with the recommendations from the SEER Program administrators, we report only those with confirmed radiation/surgery, and we acknowledge that at least some individuals who were excluded may have received these treatments [69]. We address this limitation in our sensitivity analyses described below.

These cancer groupings were created with the feedback of two practicing physicians (coauthors: L.G. and S.E.). Details on each cancer group, including ICD‐O‐3 codes and a justification for the grouping, can be found in Table 1. Although we have classified the first three groups of cancers as “fertility‐impacting,” we acknowledge that all cancers could impact fertility, especially when combined with specific types of chemotherapy. Importantly, the conceptual groupings do include some cancers that are predominantly diagnosed and experienced in childhood, including leukemia, lymphoma, and brain and central nervous system cancers. However, such cancers are not always specifically identified in the ICD‐O‐3 codes. The SEER documentation provides further information on how the ICD‐O‐3 codes overlap with the International Classification of Childhood Cancer, Third Edition (ICCC‐3) [77].

**TABLE 1:** Description and brief justification of conceptual grouping of cancer types.

	Description	Justification	ICD‐O‐3 site codes^a^	
Group 1 Cancers of Reproductive Organs (“Reproductive Cancers”)	Cancers of the reproductive organs are included in this category	Impact fertility due to location^b^	Female genital system Cervix Uteri (C530‐C539); *Corpus and Uterus, NOS*: Corpus Uteri (C540‐C549), Uterus, NOS (C559), Ovary (C569), Vagina (C529), Vulva (C510‐C519), Other Female Genital Organs (C570‐C579, C589)	Male genital system Prostate (C619), Testis (C620‐C629), Penis (C600‐C609), Other Male Genital Organs (C630‐C639)
Group 2 Proximal Cancers with Radiation/Surgery (“Proximal Plus Cancers”)	These cancers are those which are not part of the reproductive system, but their location and primary treatment by radiation or surgery may pose a risk to patients’ fertility^c^	Radiation to the brain, pelvis, and abdomen can inhibit sperm and testosterone production, estrogen production, and have other direct or indirect effects on reproductive ability. Further, surgery in the pelvic area (e.g., bladder, colon, prostate, rectal) can also negatively impact fertility, possibly because of scarring or other direct or indirect damage to the reproductive organs^d^	Digestive System^e^ *Colon and Rectum*: Cecum (C180), Appendix (C181), Ascending Colon (C182), Hepatic Flexure (C183), Transverse Colon (C184), Splenic Flexure (C185), Descending Colon (C186), Sigmoid Colon (C187), Large Intestine, NOS (C188‐C189, C260); R*ectum and Rectosigmoid Junction*: Rectosigmoid Junction (C199), Rectum (C209), Anus, Anal Canal and Anorectum (C210‐C212, C218); *Liver and Intrahepatic Bile Duct*: Liver (C220); Intrahepatic Bile Duct (C221)	Urinary System Site Group Urinary Bladder (C670‐C679), Kidney and Renal Pelvis (C649, C659), Ureter (C669), Other Urinary Organs (C680‐C689) Brain and Other Nervous System Site Group Brain (C710‐C719), Cranial Nerves Other Nervous System (C700‐C729) Endocrine System Site Group Thyroid (C739), Other Endocrine including Thymus (C379, C740‐C749, C750‐C759)
Group 3 Other Literature Identified Cancers (“Literature‐Identified Cancers”)^f^	These cancers do not fit into Group 1 or Group 2, but empirical studies have found these cancers are associated with an elevated risk of infertility	Empirical findings as indicated in the notes^g^	Breast (all) (C500‐C509) Bone and Joint (C400‐C419) Leukemia (all) (C420, C421, C424); Lymphocytic, Myeloid and Monocytic, and Other, including those without specific ICD‐O‐3 codes	Lymphoma^e^ *Hodgkin Lymphoma*: Hodgkin – Nodal (C024, C098‐C099, C111, C142, C379, C422, C770‐C779), Hodgkin—Extranodal (All other sites) Myeloma^h^

^a^ The system groupings and the ICD‐O‐3 codes come from the Site Recode information provided by the SEER program administrators; see: [70].

^b^ For further information and evidence see: [8, 60, 61, 62, 63, 64, 65].

^c^ Primary treatments were determined by reviewing the guidelines summarized by the National Cancer Institute for specific cancer types (here: [71]) and/or by seeking out specific practice recommendations from the American Society of Clinical Oncology (archived here: [72]).

^d^ For further information and evidence see: [62, 63, 66, 67].

^e^ Selected sites only; any site not listed is included in the residual “all other” cancer group.

^f^ This category of fertility‐impacting cancers is the most diverse and may require further refinement as detailed in the limitations section.

^g^ For evidence on breast cancer see: [60, 73]; for evidence on bone and joint cancers see: [12, 60]; for evidence on leukemia see: [8, 73, 74]; for evidence on lymphoma see: [8, 73, 75] for evidence on Myeloma see: [76].

^h^ No ICD‐O‐3 site code provided [70].

### 4.2 Covariates

Four covariates were included in our analyses. First, age was measured using the single year of age at the time of diagnosis variable, which we recoded into the following categories: under 1 year, 1–4, 5–9, 10–14, 15–19, 20–24, 35–29, 30–34, and 35–39 years. Second, year was measured using the year of the registry data. Third, sex was measured using the available binary categories of female and male. Finally, race/ethnicity was measured using the available categories in the registry dataset: Hispanic, non‐Hispanic American Indian or Alaska Native (hereafter: AIAN or Indigenous), non‐Hispanic Asian or Pacific Islanders (hereafter: API), non‐Hispanic Black (hereafter: Black), and non‐Hispanic white (hereafter: white).

### 4.3 Analytic Plan

Before beginning the analyses, we used the Case Listing feature of the SEER*Stat Program [78] to compile a list of individual cancer cases meeting the inclusion criteria. This dataset was exported, aggregated, and matched to the population counts from the SEER*Stat program by age, year, sex, and race/ethnicity. The aggregation of population‐level counts and all other analyses was carried out in Stata 17.0 [79].

Once this dataset was compiled, two sets of secondary analyses were completed. The first set of analyses was descriptive and calculated (1) incidence rates for each of the four groups of cancer overall and by age group and (2) age‐standardized cancer incidence rates by sex and race/ethnic groups. Standard demographic formulas were used for these calculations [80].

The second set of analyses used multivariable negative binomial regression with an exposure for population size to evaluate the associations between age, sex, and race/ethnicity and the incidence rates within each of the groups of cancer (e.g., stratified by cancer group). Exponentiated coefficients representing the incidence rate ratios (IRR) are presented. After conducing these stratified analyses, we used Wald‐tests to evaluate whether the sex and race/ethnicity associations observed among the three groups of fertility‐impacting cancers were statistically distinct from the associations observed for all other cancers (the residual group). Bonferroni‐adjusted *p*‐values were used for the Wald‐tests making multiple comparisons.

### 4.4 Supplemental Analyses

Three supplemental analyses were also conducted. First, given the potential for underreporting of radiation and surgery, we conducted the negative binomial regression analysis on an alternatively coded proximal cancer category, which removed the confirmation of radiation and surgery from the coding. Second, we conducted analyses comparing each of the three fertility‐impacting cancer groups to all‐site cancer in order to assess whether and to what extent associations in fertility‐impacting cancers varied from all‐site cancer, rather than just the residual category of all other cancers that were not classified as “fertility‐impacting.” Finally, literature‐identified cancers (group 3) in our typology include breast cancer. However, the prevalence and incidence of breast cancer disproportionately affect females [81, 82]. Therefore, we conducted an analysis of literature‐identified cancers excluding breast cancer and evaluated how this impacts the results reported in the main analyses.

## 5 Results

### 5.1 Descriptive Analyses

**FIGURE 1:**
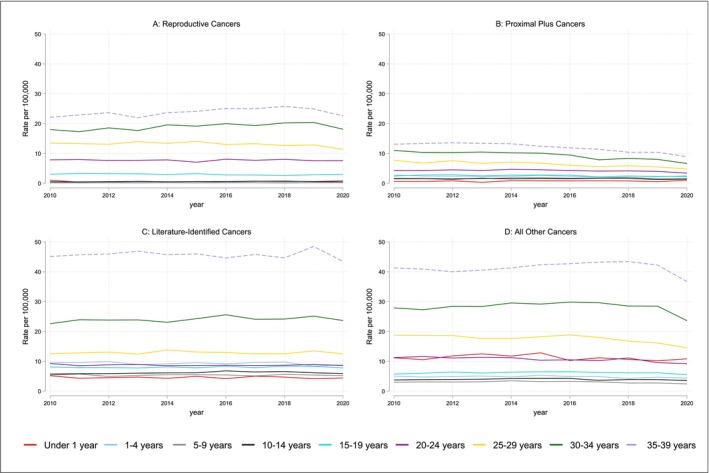
Incidence rates of (A) reproductive cancers (B) proximal plus cancers, (C) literature‐identified cancers, and (D) all other cancers by year (2010–2020) and five‐year age groups. The four groups of cancers used in this table are defined and described in detail in Table 1.

The overall age and age‐adjusted incidence rates of all four types of cancer show stratification in the rates of fertility‐impacting across sociodemographic groups (Figure 1, Table 2).

**TABLE 2:** Age‐standardized incidence rates per 100,000 by year, cancer group, and sex (Panel A) and race/ethnicity (Panel B).

	2010	2011	2012	2013	2014	2015	2016	2017	2018	2019	2020
Panel A: Sex											
Reproductive cancers											
Female	9.42	9.43	9.68	9.17	9.87	9.98	10.26	10.10	10.29	10.57	9.28
Male	7.03	7.03	7.22	7.40	7.52	7.53	7.80	7.85	8.05	7.72	7.53
Proximal plus cancers											
Female	7.50	7.25	7.50	7.13	7.23	7.04	6.52	5.89	6.08	5.57	4.83
Male	3.56	3.46	3.52	3.60	3.75	3.74	3.63	3.48	3.48	3.44	3.25
Literature‐identified cancers											
Female	20.90	21.21	21.13	21.56	21.48	21.90	22.15	22.14	22.21	23.25	22.00
Male	8.34	8.28	8.45	8.10	8.29	8.29	8.41	8.18	8.34	8.57	7.81
All other cancers											
Female	15.16	15.09	15.32	15.47	15.74	16.07	16.40	16.36	16.27	15.78	13.89
Male	14.31	14.21	14.16	14.04	14.48	14.57	14.72	14.47	14.20	14.00	12.42

Panel B: Race/ethnicity											
Reproductive cancers											
Hispanic (All races)	8.85	8.50	9.00	8.95	9.59	9.90	10.32	10.39	11.00	11.30	10.71
AIAN, NH	11.13	12.92	9.97	12.98	13.28	10.81	13.49	10.41	15.27	11.49	11.87
API, NH	5.08	5.27	5.13	5.43	5.49	5.28	5.42	5.62	5.60	6.20	5.73
Black, NH	5.45	5.74	6.34	6.01	5.73	5.90	5.99	6.56	5.86	5.99	5.52
White, NH	9.51	9.62	9.75	9.50	10.10	10.13	10.44	10.18	10.42	10.07	9.04
Proximal plus cancers											
Hispanic (All races)	4.42	4.15	4.54	4.49	4.73	4.66	4.56	4.34	4.52	4.37	3.82
AIAN, NH	5.04	6.40	6.86	4.63	7.00	6.09	5.65	5.49	5.47	5.31	2.24
API, NH	5.64	5.35	5.19	6.05	5.58	5.58	5.07	4.78	5.10	4.91	4.41
Black, NH	3.24	3.14	3.06	3.03	3.32	2.93	2.93	2.58	2.35	2.28	2.37
White, NH	6.87	6.74	6.89	6.53	6.67	6.62	6.18	5.66	5.74	5.31	4.78
Literature‐identified cancers											
Hispanic (All races)	12.66	12.86	12.96	13.53	13.36	13.76	14.21	13.94	14.19	15.04	14.25
AIAN, NH	13.25	14.88	15.75	14.66	15.60	13.23	15.50	16.34	16.53	15.72	13.46
API, NH	13.09	13.82	13.66	13.82	14.06	13.93	14.18	13.71	14.96	14.41	14.16
Black, NH	16.54	16.25	16.28	16.36	16.23	16.54	17.55	16.70	16.44	17.73	16.10
White, NH	15.92	15.94	16.04	15.78	16.02	16.24	16.09	16.36	16.27	16.97	15.79
All other cancers											
Hispanic (All races)	9.51	9.36	9.59	9.94	10.57	10.33	10.37	10.64	11.09	10.77	9.76
AIAN, NH	13.35	13.27	15.22	8.45	12.80	12.20	10.92	13.21	12.58	12.42	10.12
API, NH	8.61	9.37	9.82	9.85	9.83	10.04	11.02	10.83	10.61	10.00	8.81
Black, NH	11.69	11.19	12.45	12.19	12.12	12.56	13.11	12.69	12.44	12.46	11.43
White, NH	20.05	20.01	19.74	19.83	20.27	20.81	21.10	20.87	20.42	20.07	17.52

Abbreviations: AIAN = American Indian and Alaskan Native, NH = non‐Hispanic, NHOPI = Native Hawaiian or Other Pacific Islander.

### 5.2 Multivariable Analyses

The multivariable negative binomial regression models show a number of significant associations (Table 3). Specifically, adjusting for age, race/ethnicity, and year, the incidence rate of reproductive cancers, proximal plus cancers, and literature‐identified cancers was significantly higher for women than for men by a factor of 1.35 (95% CI: 1.25–1.47, *p* < 0.001), 1.68 (95% CI: 1.61–1.75, *p* < 0.0.001), and 1.55 (95% CI: 1.46–1.66, *p* < 0.001), respectively. By contrast, the risk of having any other cancer was significantly lower for women than for men (aIRR = 0.98, 95% CI: 0.98–0.099, *p* < 0.001).

**TABLE 3:** Negative binomial regression analyses stratified by cancer group.

	Group 1: Reproductive cancers	Group 2: Proximal plus cancers	Group 3: Literature cancers	Group 4: All others cancers
Year	1.003	0.981***	1.001	0.996
	[0.991, 1.016]	[0.975, 0.988]	[0.991, 1.011]	[0.991, 1.001]
Age (Ref = 25–29)				
Under 1 year	0.0822***	0.191***	0.542***	1.142***
	[0.0647, 0.104]	[0.160, 0.228]	[0.467, 0.628]	[1.062, 1.229]
1–4 years	0.0418***	0.666***	1.099	0.499***
	[0.0341, 0.0511]	[0.608, 0.729]	[0.965, 1.252]	[0.467, 0.534]
5–9 years	0.0256***	0.479***	0.623***	0.307***
	[0.0207, 0.0317]	[0.437, 0.526]	[0.547, 0.711]	[0.287, 0.329]
10–14 years	0.0841***	0.421***	0.741***	0.400***
	[0.0707, 0.100]	[0.384, 0.463]	[0.651, 0.845]	[0.374, 0.428]
15–19 years	0.395***	0.644***	0.942	0.612***
	[0.340, 0.458]	[0.590, 0.702]	[0.828, 1.072]	[0.574, 0.652]
25–29 years	1.645***	1.507***	1.434***	1.574***
	[1.429, 1.893]	[1.391, 1.633]	[1.262, 1.629]	[1.484, 1.670]
30–34 years	2.401***	2.248***	2.572***	2.483***
	[2.084, 2.766]	[2.078, 2.433]	[2.264, 2.922]	[2.342, 2.631]
35–39 years	3.166***	2.986***	4.823***	3.664***
	[2.744, 3.652]	[2.761, 3.229]	[4.245, 5.479]	[3.459, 3.882]
Sex (Ref = Male)				
Female	1.355***	1.678***	1.554***	0.931***
	[1.246, 1.474]	[1.607, 1.753]	[1.455, 1.660]	[0.903, 0.960]
Race/Ethnicity (Ref: White, NH)				
Hispanic (All races)	1.174**	0.708***	0.980	0.609***
	[1.053, 1.308]	[0.671, 0.748]	[0.896, 1.072]	[0.585, 0.634]
AIAN, NH	1.304***	0.880*	0.912	0.676***
	[1.127, 1.509]	[0.777, 0.997]	[0.810, 1.027]	[0.620, 0.736]
API, NH	0.629***	0.837***	0.864**	0.579***
	[0.556, 0.712]	[0.787, 0.890]	[0.788, 0.948]	[0.553, 0.607]
Black, NH	0.584***	0.486***	0.854***	0.678***
	[0.517, 0.660]	[0.456, 0.519]	[0.779, 0.935]	[0.650, 0.708]
Ln(Alpha)	0.242***	0.0523***	0.195***	0.0322***
	[0.213, 0.275]	[0.0436, 0.0627]	[0.176, 0.216]	[0.0280, 0.0369]

*Note:* Exponentiated coefficients; 95% confidence intervals in brackets.

Abbreviations: AIAN = American Indian and Alaska Native, NH = non‐Hispanic, NHOPI = Native Hawaiian or Other Pacific Islander.

* *p* < 0.05.

** *p* < 0.01.

*** *p* < 0.001.

Inequalities in the incidence rates of the three groups of fertility‐impacting cancers were also observed by race/ethnicity. First, the adjusted incidence rate (aIRR) of reproductive cancers was 1.17 (95% CI: 1.05–1.31, *p* < 0.01) times as high for the Hispanic population and 1.30 (95% CI: 1.12–1.51, *p* < 0.001) times as high for the Indigenous (AIAN) population relative to the white population, holding the other covariates constant. However, the adjusted incidence rates of reproductive cancers were lower for Black (aIRR = 0.58, 95% CI: 0.52–0.66, *p* < 0.001) and API populations (aIRR = 0.63, 95% CI: 0.56–0.71, *p* < 0.001) relative to the white population.

Second, the incidence rates of proximal plus cancers and all other cancers were significantly lower among all racial/ethnic groups relative to the white population (Table 3). Meanwhile, the incidence rates of literature‐identified cancers were statistically similar for Hispanic (aIRR = 0.98, 95% CI: 0.90–1.07, *p* > 0.05) and Indigenous (AIAN) populations (aIRR = 0.91, 95% CI: 0.81–1.03, *p* > 0.05) relative to the white population, while the incidence rates were lower for API (aIRR = 0.86, 95% CI: 0. 0.79–0.95, *p* < 0.01) and Black populations (aIRR = 0.85, 95% CI: 0.78–0.95, *p* < 0.001) relative to the white population.

### 5.3 Wald‐Test Results: Comparison Between Fertility‐Impacting Cancers and All Other Cancers

We utilized Wald tests to compare the model coefficients on sex and race/ethnicity across the cancer group stratified models. We focused specifically on comparing the three groups of fertility‐impacting cancers to the residual all other cancers group (Table 4).

**TABLE 4:** Wald test comparing model coefficients for sex and race/ethnicity between fertility‐impacting cancers and all other types of cancer.

	Reproductive cancers versus all other cancers		Proximal plus cancers versus all other cancers		Literature cancers versus all other cancers	
	*χ* ^2^	*p* ^a^	*χ* ^2^	*p*	*χ* ^2^	*p*
Sex (df = 1)	63.98	< 0.001	533.16	< 0.001	211.36	< 0.001
Race/Ethnicity				< 0.001		< 0.001
Hispanic (All races)	126.37	< 0.001	24.52	< 0.001	185.39	< 0.001
AIAN, NH	66.46	< 0.001	14.86	< 0.001	18.32	< 0.001
API, NH	1.83	0.706	112.20	< 0.001	76.32	< 0.001
Black, NH	3.91	0.193	67.85	< 0.001	22.68	< 0.001
Overall (df = 4)	229.79	< 0.001	268.15	< 0.001	198.09	< 0.001

Abbreviations: AIAN = American Indian and Alaskan Native, df = degrees of freedom, NH = non‐Hispanic, NHOPI = Native Hawaiian or Other Pacific Islander.

^a^ Bonferroni adjusted *p*‐values are presented for the Wald tests on the race/ethnicity coefficients.

First, for reproductive cancers, the coefficient on sex differed significantly between reproductive cancers and all other cancers (*χ* ^2^ = 63.98, *p* < 0.001). Next, when analyzing race/ethnicity, the incidence rate ratios for reproductive cancers and all other cancers significantly differed overall (*χ* ^2^ = 229.79, *p* < 0.001) with pairwise tests showing significant differences for Hispanic (*χ* ^2^ = 126.37, *p* < 0.001) and Indigenous (*χ* ^2^ = 66.46, *p* < 0.001) populations relative to white populations.

Second, for proximal plus cancers, the coefficient for sex differed significantly from all other types of cancers (*χ* ^2^ = 533.16, *p* < 0.001). In addition, there was an overall difference in the race/ethnicity coefficients when comparing proximal plus cancers to all other cancers (*χ* ^2^ = 268.15, *p* < 0.001). Pairwise comparisons showed that there were significant differences for Hispanic (*χ* ^2^ = 24.52, p < 0.001), Indigenous (*χ* ^2^ = 14.86, *p* < 0.01), Asian (*χ* ^2^ = 112.20, *p* < 0.01), and Black (*χ* ^2^ = 67.85, *p* < 0.001) populations when comparing proximal plus cancers and all other types of cancer relative to the white population.

Finally, for literature‐identified cancers, the coefficient for sex differed significantly from all other cancers (*χ* ^2^ = 211.36, *p* < 0.001). As observed above, there was an overall difference in the race/ethnicity coefficients when comparing literature‐identified cancers to all other cancers (*χ* ^2^ = 198.09, *p* < 0.001). Similarly, pairwise comparisons for literature‐identified cancers and all other cancers showed significant differences for all racial/ethnic groups (Hispanic: *χ* ^2^ = 185.39, *p* < 0.001; Indigenous: *χ* ^2^ = 18.32, *p* < 0.001; Asian: *χ* ^2^ = 76.32, *p* < 0.01; Black *χ* ^2^ = 22.68, *p* < 0.001).

### 5.4 Supplemental Analyses

In the first supplemental analysis, we conducted a multivariable negative binomial regression using a revised version of the proximal plus cancers group, which excluded confirmation of radiation/surgery as an inclusion criterion. The results of these analyses were similar to the main analyses with two exceptions (Table S1). First, there was a positive association between the incidence rate of proximal cancers and year, which was not observed in the main findings (aIRR = 1.006, 95% CI: 1.002, 1.011, *p* < 0.01). Second, there was no statistical difference between the incidence rates of this group of cancers between white and Indigenous populations, which was observed in the main findings.

The second set of supplemental analyses repeated the Wald tests above but compared the coefficients from the fertility‐impacting cancers to the coefficients from a negative binomial regression analysis of all‐site cancer (Table S1 reports the negative binomial regression results, and Table S2 reports the results of the Wald tests). The results show similarities and differences from those presented in the main analyses. When comparing reproductive cancers to all‐site cancers, we found the coefficient for sex did not differ statistically (*χ* ^2^ = 1.85, *p* > 0.05) unlike the comparison to all other cancers in the main analyses. However, similar to the main analyses, the incident rate ratio for reproductive cancers and all‐site cancers also differed significantly overall for race/ethnicity (*χ* ^2^ = 177.52, *p* < 0.001) with pairwise comparisons showing differences between the white population and Hispanic (*χ* ^2^ = 52.55, *p* < 0.001), Indigenous (*χ* ^2^ = 37.40, p < 0.001), and Black (*χ* ^2^ = 11.88, *p* < 0.05) populations.

Next, when comparing proximal plus cancers to all‐site cancer, we found the coefficients for sex did differ significantly (*χ* ^2^ = 167.20, *p* < 0.001), similar to findings in the main analyses. In addition, there was a significant overall difference in the race/ethnicity coefficients when comparing proximal plus cancers to the all‐site cancer group (*χ* ^2^ = 252.74, *p* < 0.001) with significant pairwise comparisons for Hispanic (*χ* ^2^ = 34.89, *p* < 0.001), API (*χ* ^2^ = 41.08, *p* < 0.001), and Black (*χ* ^2^ = 131.80, *p* < 0.001) populations.

Finally, when comparing to literature‐identified cancers, we found that the coefficients for sex did statistically differ (*χ* ^2^ = 59.34, *p* < 0.001), similar to the findings in the main analyses. As observed above, there was an overall difference in the race/ethnicity coefficients when comparing literature‐identified cancers to all‐site cancer (*χ* ^2^ = 91.19, *p* < 0.001) with pairwise comparisons showing differences for Hispanic (*χ* ^2^ = 64.81, *p* < 0.001), API (*χ* ^2^ = 45.34, *p* < 0.001), and Black (*χ* ^2^ = 29.35, *p* < 0.001) populations.

The difference in these findings from the main analyses is not necessarily surprising as the residual category of “all other cancers” (group 4) is distinct from the “all‐site cancer” category. However, these analyses demonstrate that the patterning in the fertility‐impacting cancers differs from both the residual other cancers and any cancer categories.

In the final supplemental analysis, we assessed the patterning in the literature‐identified cancers (group 3) excluding breast cancer (Table S3). The results of this supplemental analysis show three differences from the main findings. First, and most notably, when excluding breast cancers, the incidence rate of literature‐identified cancers was lower among females than males (aIRR = 0.84, 95% CI: 0.82, 0.96, *p* < 0.001). Second, there were also differences in the direction and statistical significance of coefficients by age. Finally, while the statistical significance and direction of effects were similar by race/ethnicity, a Wald test revealed the coefficients for API populations differed between the two models (*χ* ^2^ = 7.13, *p* < 0.05). Overall, this supplemental analysis confirms that the sex effects observed in the main findings were being driven by breast cancer and suggests there may be slightly different associations between incidence rates of these types of cancers by race/ethnicity when excluding or including breast cancer.

## 6 Discussion

Infertility can be a late effect that is of particular concern for younger cancer patients. In this study analyzing incident rates of three groups of fertility‐impacting cancers (1) cancers of the reproductive organs, (2) cancers proximate to the reproductive organs or central to hormone production related to fertility, and (3) other cancers that have been associated with infertility in previous research, we found differences in the sex and racial/ethnic patterning of these cancers both within fertility‐impacting cancer groups and between fertility‐impacting cancers and the residual “all other cancer” group. Our second supplemental analysis also confirmed the patterns observed for fertility‐impacting cancers differed from all‐site cancers. Thus, the findings of this study suggest that the burden of fertility‐impacting cancers may differ from other types of cancer.

Specifically, the results of this study showed women had higher incidence rates of all three groups of fertility‐impacting cancers. Such findings could be explained by biological or hormonal differences, more regular preventative care contact with health care systems resulting in increased screening and detection, and/or differential care‐seeking behaviors among women and men [83, 84, 85, 86, 87, 88]. Notably, the patterns observed in fertility‐impacting cancers differs from the residual “all other cancers,” which suggests that part of the higher overall incidence rate of all‐site cancer among women may be driven by these groups of fertility‐impacting cancers.

Additionally, this study found that the racial/ethnic patterning in the three different groups of fertility‐impacting cancers often differed from the patterning observed in the residual “all other cancers group.” Specifically, we found the incidence rate of cancers of the reproductive organs was higher among Hispanic and Indigenous populations and lower among Black and Asian/Pacific Islander populations relative to non‐Hispanic white populations. Meanwhile, incidence rates tended to be similar or lower among populations of color relative to non‐Hispanic white populations for the other two groups of fertility‐impacting cancers.

The variation observed by race/ethnicity in incidence rates suggests that different mechanisms may be at work across the different fertility‐impacting cancers. For instance, the high rates of reproductive cancers among Indigenous and Hispanic populations may be driven by the high rates of cervical cancer among these populations documented in prior studies [89, 90, 91, 92, 93, 94, 95], which scholars have connected to mechanisms such as low human papillomavirus vaccine uptake related to a complex web of historical, economic, infrastructural, and social injustices [89, 90, 95]. Further, some evidence has found Hispanic populations are more likely to receive screening for cervical cancer, which may also increase detection and, thus, incidence rates [96]. As a result, it is possible that differential exposure to risk and/or screening engagement is driving these associations.

By contrast, the lower incidence rates observed among populations of color in the proximal plus and literature‐identified fertility‐impacting cancer groups may be due to lower screening rates or lower rates of receipt of treatment among populations of color. For instance, screening mammography is lower among Black and Hispanic populations [97], which might result in a lower incidence rate of breast cancer due to lack of detection. Similarly, receipt of cancer treatment is lower among populations of color [98], which may contribute to the appearance of lower incidence rates of the group of fertility‐impacting cancers that involved radiation/surgery in areas proximate to reproduction (though the sensitivity analysis suggested these lower incidence rates persisted even when removing confirmed receipt of treatment). Overall, however, it is likely that a combination of mechanisms contributes to these findings. The data used in this study do not allow these mechanisms to be directly tested. Thus, further research is needed to clarify why the association between race/ethnicity and different fertility‐impacting cancers varies both from one another and from other cancer incidence rates.

### 6.1 Limitations

This study has several key limitations. First, due to data availability, this study does not account for SES or other sociodemographic characteristics beyond age, race/ethnicity, and sex. Future research could investigate these associations with survey data or registry data with more information on SES. Second, and relatedly, the study does not test the mechanisms of the inequalities observed. Third, the SEER registry, though large, is not nationally representative, and the patterns observed in this study may be influenced by the specific registries included.

Fourth, our analyses conceptually grouped different types of fertility‐impacting cancers together, but such a grouping may disguise inequalities within the cancer groups (e.g., between cervical cancer and uterine cancer) and may, therefore, have contributed to the mixed findings observed. Relatedly, as observed in the final supplemental analysis, conceptual groupings that include prevalent cancers that disproportionately affect one sociodemographic may have a substantive impact on the results. Therefore, the use of these conceptual groups should be considered carefully in light of the goals of the research. In the case of the present study, our focus was on understanding the general trends and burdens of fertility‐impacting cancers across groups. Therefore, the results of the main analyses, including breast cancer as a literature‐identified cancer, are valid for our purposes as they demonstrate a higher burden of these types of cancers among women (even if it is being driven by one specific type of cancer). For other research purposes, this conclusion may not reflect the goals of the paper, and it is possible that further refinement to the literature‐identified cancers could improve the precision of the typology.

Finally, it is not clear whether or to what extent the patterns observed in these analyses will apply to other contexts with differing health care systems and social stratification. Future research could explore this issue by replicating our analyses using cancer registries from other countries.

### 6.2 Implications for Research and Practice

Despite these limitations, this work has important implications for research and practice. First, the results point to the continued need for the screening of fertility‐impacting cancers. That is, one of the potential reasons for differential incidence rates by sociodemographic groups may be engagement in screening, which could be addressed by reducing inequalities in cancer screenings. Second, the results confirm the importance of disaggregating all‐site cancer incidence rates when researching disparities, especially when considering the potential unmet need for fertility care. Finally, the results show misalignment between the incidence of fertility‐impacting cancers and fertility preservation referrals.

Specifically, the aforementioned research shows lower rates of referral to FP among cancer patients who are women, people of color, populations with lower SES, and those without children [10, 20, 23, 24, 25, 26, 99]. Yet, our findings show higher incidence rates of fertility‐impacting cancers for women, and in some groups, people of color. This suggests that there is some misalignment between FP referrals and the incidence of fertility‐impacting cancers. Yet, it is also important to note that other factors likely contribute to the observed inequalities, including, though not limited to, the likelihood of being nulliparous or without children at the time of a diagnosis of fertility‐impacting cancers (due to differences in the timing of first births across sex, SES and racial‐ethnic groups in the U.S. [100]). In other words, referrals may not be made to some groups due to factors beyond the incidence of fertility‐impacting cancers. Further, access and use of FP after a referral may also vary based on economic, geographic, or social factors as observed in prior research [28, 29, 30, 31, 32, 33, 35, 101].

As a result, addressing these inequalities will require a range of interventions at the provider, health system, and societal levels. Our results also confirm that it is not differential need (e.g., differential incidence rates of fertility‐impacting cancers) that should explain such inequalities. Thus, efforts to improve FP referral inequalities are still needed; two specific and actionable interventions based on previous research for practice include: (1) creating standardized, accessible educational materials to address prior work showing patients report uneven quality and presence of information about infertility during cancer care [102, 103, 104, 105, 106] and (2) establishing clear, health‐system‐level protocols for FP referrals that do not rely upon individual practice, given the research among oncologists has found referrals to be uneven and almost ad‐hoc in nature [21, 22]. Such interventions do not address inequality in access to FP, but they may ensure patients across sociodemographic groups have more even knowledge and the opportunity for consultation with fertility specialists, which may increase equity in the ability to use fertility treatments later in life (among those who utilize FP) as well as satisfaction with care and quality of life [19].

## 7 Conclusion

Overall, this study has shown that the sex and racial/ethnic patterning of fertility‐impacting cancers differs from other types of cancers. When considering cancers of the reproductive organs, women and Hispanic and Indigenous populations are disproportionately affected. Yet, these groups often receive referrals to fertility preservation less often, showing a potential misalignment between needs and resources. Thus, the results of this study point to the ongoing need to screen for fertility‐impacting cancers, to understand the social and structural mechanisms contributing to these inequalities, and to ensure comprehensive fertility counseling is provided to all cancer patients who may benefit from these services.

## Author Contributions

**Katherine I. Tierney:** conceptualization (lead), formal analysis (lead), funding acquisition (lead), investigation (lead), methodology (lead), project administration (lead), supervision (lead), writing – original draft (lead), writing – review and editing (lead). **Jennifer Therrien:** formal analysis (supporting), writing – original draft (supporting), writing – review and editing (supporting). **Stephanie Ellwood:** conceptualization (supporting), validation (supporting), writing – review and editing (supporting). **Lisa Graves:** conceptualization (supporting), validation (supporting), writing – review and editing (supporting).

## Consent

The authors have nothing to report.

## Conflicts of Interest

The authors declare no conflicts of interest.

## Supporting information


Table S1.

## Data Availability

The data used for this study are publicly available.
