# Supplementary material for: Inequalities in Fertility‐Impacting Cancer Incidence Among Young Populations in the United States
Source: Cancer Med. 2025 Apr 6;14(7):e70797. doi: 10.1002/cam4.70797 (PMC11973136; doi:10.1002/cam4.70797)
Supplement: Supplementary file 1 — Table S1. [file CAM4-14-e70797-s001.docx]

**Supplemental Table 1**: **Negative Binomial Regression between Year, Age, Sex, and Race/Ethnicity for Alternatively Coded Proximal Cancers and for All-Site Cancer**

|  | **Proximal Cancers with or Without Treatment Confirmed** | **All-Site Cancer** |
| --- | --- | --- |
|  |  |  |
| **Year** | 1.006** | 1.002 |
|  | [1.002,1.011] | [0.997,1.006] |
| **Age (Ref = 25-29)** |  |  |
| Under 1 year | 0.772*** | 0.697*** |
|  | [0.722,0.827] | [0.654,0.743] |
| 1-4 years | 0.591*** | 0.573*** |
|  | [0.557,0.627] | [0.541,0.608] |
| 5-9 years | 0.398*** | 0.359*** |
|  | [0.375,0.423] | [0.338,0.381] |
| 10-14 years | 0.450*** | 0.433*** |
|  | [0.424,0.477] | [0.408,0.459] |
| 15-19 years | 0.689*** | 0.671*** |
|  | [0.651,0.730] | [0.633,0.710] |
| 25-29 years | 1.557*** | 1.563*** |
|  | [1.475,1.644] | [1.478,1.652] |
| 30-34 years | 2.411*** | 2.492*** |
|  | [2.285,2.544] | [2.358,2.634] |
| 35-39 years | 3.500*** | 3.822*** |
|  | [3.318,3.691] | [3.617,4.039] |
| **Sex (Ref = Male)** |  |  |
| Female | 1.499*** | 1.284*** |
|  | [1.458,1.541] | [1.249,1.320] |
| **Race/Ethnicity (Ref: White, NH)** |  |  |
| Hispanic (All Races) | 0.741*** | 0.813*** |
|  | [0.714,0.769] | [0.782,0.846] |
| AIAN, NH | 0.961 | 0.911*** |
|  | [0.901,1.025] | [0.863,0.962] |
| API, NH | 0.716*** | 0.713*** |
|  | [0.687,0.746] | [0.684,0.743] |
| Black, NH | 0.722*** | 0.723*** |
|  | [0.694,0.751] | [0.695,0.753] |
| **Ln(Alpha)** | 0.0302*** | 0.0375*** |
|  | [0.0264,0.0345] | [0.0337,0.0418] |

*Abbreviations*: NH= non-Hispanic, AIAN= American Indian and Alaskan Native, NHOPI= Native Hawaiian or Other Pacific Islander

Exponentiated coefficients; 95% confidence intervals in brackets

* p<0.05, ** p<0.01, *** p<0.001

**Supplemental Table 2: Wald Test Comparing Model Coefficients for Sex and Race/Ethnicity between Fertility-Impacting Cancers and All-Site Cancer**

|  | **Reproductive Cancers vs All-Site Cancers** | | **Proximal Plus Cancers vs All-Site Cancers** | | **Literature Cancers vs All-Site Cancers** | |
| --- | --- | --- | --- | --- | --- | --- |
|  | *Χ^2^* | *p-value^1^* | *Χ^2^* | *p-value* | *Χ^2^* | *p-value* |
| **Sex (df=1)** | 1.85 | 0.174 | 167.20 | >0.001 | 59.34 | <0.001 |
| **Race/Ethnicity** |  |  |  | >0.001 |  | <0.001 |
| Hispanic (All Races) | 52.55 | <0.001 | 34.89 | >0.001 | 64.81 | <0.001 |
| AIAN, NH | 37.40 | <0.001 | 0.38 | 1.000 | 0.00 | 1.000 |
| API, NH | 6.13 | 0.053 | 41.08 | >0.001 | 45.34 | <0.001 |
| Black, NH | 11.88 | 0.002 | 131.80 | >0.001 | 29.35 | <0.001 |
| *Overall (df=4)* | 177.52 | <0.001 | 252.74 | >0.001 | 91.19 | <0.001 |

*Abbreviations*: NH= non-Hispanic, AIAN= American Indian and Alaskan Native, NHOPI= Native Hawaiian or Other Pacific Islander; df = degrees of freedom

Notes:

1. Bonferroni adjusted p-values presented for the Wald tests on the race/ethnicity coefficients.

**Supplemental Table 3**: **Negative Binomial Regression between Year, Age, Sex, and Race/Ethnicity and Group 3 Cancer Including and Excluding Breast Cancer**

|  | **Main Coding: Group 3 Cancers, Including Breast Cancer** | **Sensitivity Coding: Group 3 Cancers, Excluding Breast Cancer** |
| --- | --- | --- |
|  |  |  |
| **Year** | 1.001 | 0.998 |
|  | [0.991,1.011] | [0.994,1.003] |
| **Age (Ref = 25-29)** |  |  |
| Under 1 year | [0.467,0.628] | [0.550,0.644] |
|  | 1.099 | 1.188*** |
| 1-4 years | [0.965,1.252] | [1.128,1.252] |
|  | 0.623*** | 0.686*** |
| 5-9 years | [0.547,0.711] | [0.650,0.724] |
|  | 0.741*** | 0.802*** |
| 10-14 years | [0.651,0.845] | [0.760,0.845] |
|  | 0.942 | 1.008 |
| 15-19 years | [0.828,1.072] | [0.958,1.062] |
|  | [0.467,0.628] | [0.550,0.644] |
| 25-29 years | 1.434*** | 0.98 |
|  | [1.262,1.629] | [0.931,1.031] |
| 30-34 years | 2.572*** | 1.045 |
|  | [2.264,2.922] | [0.993,1.100] |
| 35-39 years | 4.823*** | 1.210*** |
|  | [4.245,5.479] | [1.150,1.273] |
| **Sex (Ref = Male)** |  |  |
| Female | 1.554*** | 0.836*** |
|  | [1.455,1.660] | [0.815,0.858] |
| **Race/Ethnicity (Ref: White, NH)** |  |  |
| Hispanic (All Races) | 0.98 | 0.984 |
|  | [0.896,1.072] | [0.953,1.016] |
| AIAN, NH | 0.912 | 0.943 |
|  | [0.810,1.027] | [0.857,1.039] |
| API, NH | 0.864** | 0.772*** |
|  | [0.788,0.948] | [0.741,0.804] |
| Black, NH | 0.854*** | 0.837*** |
|  | [0.779,0.935] | [0.807,0.868] |
| **Ln(Alpha)** | 0.195*** | 0.0175*** |
|  | [0.176,0.216] | [0.0143,0.0214] |

*Abbreviations*: NH= non-Hispanic, AIAN= American Indian and Alaskan Native, NHOPI= Native Hawaiian or Other Pacific Islander

Exponentiated coefficients; 95% confidence intervals in brackets

* p<0.05, ** p<0.01, *** p<0.001
